# Impaired interoception in Colombian victims of armed conflict with PTSD: a preliminary HEP study

**DOI:** 10.3389/fpsyg.2025.1567574

**Published:** 2025-04-25

**Authors:** Eduar Herrera, Daniela Gutierrez-Sterling, Alvaro Barrera-Ocampo, Juliana Orozco Jaramillo, Hernando Santamaría-García, Agustina Birba

**Affiliations:** ^1^Departamento de Estudios Psicológicos, Universidad Icesi, Cali, Colombia; ^2^Facultad de Psicología, Universidad Pontificia Bolivariana, Palmira, Colombia; ^3^Grupo Natura, Facultad de Ingeniería, Diseño y Ciencias Aplicadas, Departamento de Ciencias Farmacéuticas y Químicas, Universidad Icesi, Cali, Colombia; ^4^Departamento de Ciencia Jurídica y Política, Universidad Javeriana, Cali, Colombia; ^5^PhD Program of Neuroscience, Pontificia Universidad Javeriana, Hospital San Ignacio, Center for Memory and Cognition, Intellectus, Bogotá, Colombia; ^6^Cognitive Neuroscience Center (CNC), Universidad de San Andrés, Buenos Aires, Argentina

**Keywords:** interoception, violence, posttraumatic stress disorder, emotion recognition, heartbeat evoked cortical potential amplitude

## Abstract

Individuals who have been exposed to violence are at high risk of developing mental health problems, particularly posttraumatic stress disorder (PTSD). A prominent example is the experience of Colombia, which has suffered systemic violence for more than half a century. Subjects with trauma-related disorders have problems regulating their emotions and facial emotion recognition (FER), a phenomenon that can be explained from a biological perspective by interoception. We conducted an experimental study using the heartbeat-evoked cortical potential amplitude (HEP) to determine the differences in FER and interoceptive priming in victims of armed conflict in Colombia with PTSD, complex posttraumatic stress disorder (CPTSD), and a control group. The results of behavioral studies indicate that individuals with PTSD and CPTSD exhibit impairments in interoceptive accuracy and deficits in the FER task. Compared with those in both the control and PTSD groups, the group of CPTSD victims demonstrated a decline in FER performance following interoceptive priming relative to exteroceptive priming. At the brain level, compared with controls, individuals with CPTSD presented a reduced amplitude of the HEP in the frontocentral regions during interoceptive processing. Significant differences were observed between the CPTSD and PTSD groups in the right frontal–lateral region during interoceptive priming. Our findings suggest alterations in FER interoception and HEP attenuation in armed conflict victims with PTSD and CPTSD. These results highlight the importance of interoception tasks in understanding the neurobiological mechanisms underlying emotional regulation and recognition in populations exposed to war trauma, and they may offer potential therapeutic strategies and targets for PTSD.

## 1 Introduction

The violence, displacement, and war stemming from over 60 years of armed conflict in Colombia have led to widespread mental disorders affecting multiple generations (Pérez-Olmos et al., 2005; Martínez et al., 2016; Ramírez-Giraldo et al., 2017). Specifically, exposure to war has been linked to mental health issues such as PTSD (Angel, 2016). Research on the effects of war in Colombia highlighted the heightened risk of developing PTSD among victims (Campo-Arias et al., 2014; Hewitt Ramírez et al., 2014; Marroquín Rivera et al., 2020; Charles, 2022). Recently, a significant portion of war victims have presented CPTSD, a new category of PTSD from the International Classification of Disease (ICD)-11 that includes the core PTSD symptoms and three symptom clusters of (Pérez-Olmos et al., 2005) affective dysregulation, (Martínez et al., 2016) negative self-concept and (Ramírez-Giraldo et al., 2017) disturbed relationships, which collectively represent “disturbances in self-organization” (DSO) (Karatzias et al., 2023; Maercker et al., 2013; Organization WH, 2018). PTSD-related disorders are associated with difficulties in cognitive processes, including emotional regulation, FER, and interoceptive processes (which involve the brain processes that receive, process, and send information about internal body states) (Tanaka et al., 2023; Motomura et al., 2019; Terasawa et al., 2021), across different populations (Castro-Vale et al., 2020; Passardi et al., 2019; Couette et al., 2022), including war victims (Poljac et al., 2011; Gebhardt et al., 2017; Mazza et al., 2012). FER alterations are associated with interoception deficits. Interoception involves several cognitive and behavioral biological markers (Garfinkel et al., 2021; Garfinkel and Critchley, 2016; Suksasilp and Garfinkel, 2022) and is fundamental to homeostasis and allostatic processes (Santamaría-García et al., 2024). Interceptive and FER dysfunctions are strongly related and have been implicated in multiple physical and psychological disorders (Bonaz et al., 2021; Garfinkel and Critchley, 2016; Santamaría-García et al., 2024; Barrett and Simmons, 2015). Neurobiological models, including the somatic marker hypothesis (SMH) (Damsio, 1994), suggest that perceiving internal bodily signals and emotional awareness share a close physiological relationship and significantly influence decision-making processes. Previous research has associated interoception—and specifically heartbeat-evoked potentials (HEP)—with emotional awareness (Damasio, 1999) and autonomic nervous system (ANS) functioning within the framework of the SMH. Similarly, the Somatovisceral Afference Model of Emotion (SAME) (Cacioppo et al., 1992) supports this connection by proposing that emotions result from the brain’s interpretation of interoceptive signals, particularly those processed within the insular cortex. Alterations in these interoceptive processes contribute to deficits in emotional recognition and interoceptive sensitivity frequently observed in PTSD.

Interoception processes are altered in PTSD patients through two main mechanisms (Putica et al., 2024). The first involves dysregulations in physiological stress axes (Yehuda et al., 2015) and impairments in sympathetic and parasympathetic control that influence cardiac signaling related to abnormal cortical representation of cardiac interoceptive signals (Flasbeck et al., 2020; Ge et al., 2020). The second mechanism involves altered neurophysiological bases of mind–body interactions with altered functions of the insula, medial prefrontal cortex, anterior cingulate cortex and amygdala in individuals with PTSD (Fine et al., 2023; Lieberman et al., 2023; Harricharan et al., 2020; Nicholson et al., 2022; Akiki et al., 2017; McCurry et al., 2020; Nicholson et al., 2020; Nicholson et al., 2017; Beutler et al., 2022; Reinhardt et al., 2020). Electrophysiologically, HEP serves as a key interoceptive frontal cortical marker. This marker monitors cardiac activity, which is regulated by attention to the heartbeat, with peak modulation occurring between 200 and 500 ms after the R wave (Pollatos et al., 2016; Pollatos and Schandry, 2004; Pollatos and Schandry, 2008). The HEP amplitude reflects interoceptive accuracy (Pollatos and Schandry, 2004; Yuan et al., 2007) and characterizes different interoceptive deficits (Couto et al., 2015; Migeot et al., 2023; Fittipaldi et al., 2020) in various neuropsychiatric diseases (Abrevaya et al., 2020), such as the behavioral variant of frontotemporal dementia (Birba et al., 2022; Migeot et al., 2022; Salamone et al., 2021), amyotrophic lateral sclerosis (Moretta et al., 2022), autism spectrum disorder (Hatfield et al., 2019), affective disorders (Grossi et al., 2017), eating disorders (Donofry et al., 2016; Cambi et al., 2024), emotional dysregulation (Pang et al., 2019), chronic stress (Shatrova et al., 2024), PTSD and CPTSD (Simmons et al., 2009).

PTSD and CPTSD exhibit a range of interoceptive disturbances, including body dissociation, reduced interoceptive signal processing (Mueller et al., 2015; Schmitz et al., 2021), varying levels of interoceptive accuracy, from lowered to normal (Hart et al., 2013; Eggart et al., 2019), and reduced interoceptive sensitivity (Schmitz et al., 2021; Flasinski et al., 2020). To the best of our knowledge, no study has investigated the relationship between FER and interoception in war victims with PTSD and CPTSD. Against this background, this study evaluated FER through interoceptive priming tasks (Salamone et al., 2021; Schneider et al., 2020) in victims of the Colombian conflict with PTSD and CPTSD. The affect–emotion model of the interoceptive priming task assumes that the initial classification of stimuli by valence occurs prior to cognitive analysis during the early stages of perceptual processing (Barrett and Bar, 2009). Given this framework, electrophysiological correlates of this task show selective disruptions in interoceptive regions and significant differences in emotion recognition in neurodegenerative populations (Salamone et al., 2021). Studying FER and interception in victims with early exposure to war with PTSD and CPTSD is crucial for understanding the impact of war on interception and its neurophysiological mechanisms. Given that models such as the SMH and SAME underscore the relationship between interoception and emotional regulation, investigating these processes in victims of armed conflict diagnosed with PTSD and CPTSD is crucial. Such research can provide valuable insights into the neurobiological mechanisms underlying emotional regulation and interoceptive deficits among populations exposed to violence.

Considering the established connection between emotion processing and interoception (Adolfi et al., 2017) and the interoceptive and emotional dysfunctions in PTSD patients, as well as the altered emotion in victims of armed conflict in Colombia (Trujillo et al., 2019), this study could provide a relevant model for evaluating interoceptive priming in PTSD and CPTSD (Reinhardt et al., 2020; Richter and Ibáñez, 2021). We hypothesized that participants who were victims of armed conflict with PTSD and CPTSD symptoms would perform worse in FER than the control group. Additionally, it was expected that the victim group (PTSD-CPTSD) would present patterns associated with lower rates of FER and interoceptive accuracy than the control group, possibly due to alterations in interoceptive pathways. Finally, we expected victims with CPTSD to show differences in the amplitude of the HEP in frontocentral regions during interoceptive processing with higher amplitudes, unlike the control groups with lower amplitudes.

## 2 Materials and methods

### 2.1 Participants

The study included 44 medication-free participants with a mean age of 52.82 (SD = 8.11), 31 females (70.5%) and 13 males (29.5%) with 12 years of schooling (Mean = 12.32, SD = 4.52) were distributed in 3 groups —17 healthy controls (CG), 19 victims (PTSD), and 8 victims with CPTSD—using the International Trauma Questionnaire (ITQ), which allows the identification of the distinction between PTSD and CPTSD symptomatology (Table 1 and Supplementary Table 1). The sample size was calculated with an A-priori analysis in G-Power 3.1 software. Owing to the design of this study and population, a moderate effect size of *F* = 0.25, a power of 0.95 and a *p* = 0.05 were used, and establishing a total sample of 36 subjects is adequate to determine the estimated effects. The participants were contacted through nonprofit organizations and community leaders supported by the Victims Unit of the Department of Valle del Cauca-Colombia. The victims had reported previously (Organization WH, 2018) and were extensively assessed in previous studies (Soleimani et al., 2024; Yousefi and Abdoli, 2024; Bressington et al., 2024). All participants underwent cognitive assessment using standardized instruments (Nasreddine et al., 2005; Torralva et al., 2009) and specialized questionnaires (Supplementary material 1.2, Clinical measures). The exclusion criteria included a history of mental disorders or active psychiatric illness, illiteracy, and failure to sign the informed consent form (Supplementary material 1.2, Clinical measures). The research protocol was approved by the Ethics Committee of ICESI University, and informed consent was obtained in accordance with the Declaration of Helsinki.

**Table 1:** Demographic and neuropsychological results.

Demographics	CPTSD	PTSD	CG	Statistics	CG vs. PTSD	CPTSD vs. PTSD	CG vs. CPTSD
Gender	8:1	13:9	12:5	*X*^2^ = 2.68			
				*p* = 0.26			
Age	50.8 (7.86)	52 (8.69)	54.9 (7.18)	*F* = 0.95	*p* = 0.50	*p* = 0.92	*p* = 0.44
				*p* = 0.39			
				$\eta_{p}^{2}$ = 0.05			
Education	10.7 (5.89)	12.2 (4.19)	14.05 (3.64)	*F* = 1.78	*p* = 0.42	*p* = 0.66	*p* = 0.17
				*p* = 0.17			
				$\eta_{p}^{2}$ = 0.11			
MoCA	20.4 (4.58)	21.5 (4.22)	25.8 (2.75)	*F* = 8.40	*p* = < 0.01**	*p* = 0.76	*p* = < 0.01**
				*p* = <0.001***			
				$\eta_{p}^{2}$ = 0.32			
HSCL-58	1.77 (0.50)	0.76 (0.57)	0.57 (0.42)	*F* = 15.6	*p* = 0.50	*p* = 0.001 ***	*p* = < 0.0001***
				*p* = <0.0001***			
				$\eta_{p}^{2}$ = 0.43			
Executive functions							
IFS	18.7 (5.33)	17.6 (4.82)	24.6 (1.81)	*F* = 13.23	*p* = < 0.001***	*p* = 0.79	*p* = < 0.01**
				*p* = <0.001***			
				$\eta_{p}^{2}$ = 0.48			

The values reported correspond to the mean and standard deviation (SD). Effect sizes were calculated through partial eta-squared ( $\eta_{p}^{2}$ ). Demographic and clinical data were evaluated by ANOVAs, except for gender which was analyzed by Pearson’s *χ*^2^. **Variable with significant differences (*p* ≤ 0.05). ***Variable with significant differences (*p* ≤ 0.005).

### 2.2 Measures and procedure

#### 2.2.1 Interoceptive priming task

The interoceptive priming task consisted of two phases. The first one, the priming task, consisted of an interoceptive or exteroceptive block. In this phase, the participants were positioned in front of a 16-inch monitor screen and a keyboard. They were required to press the “Z” key while imagining/feeling their heartbeat without feedback for 2 min (interoceptive condition) or following a recorded heartbeat (exteroceptive condition), using the same procedure performed by Salamone, Legaz (Salamone et al., 2021) with a standardized heartbeat detection task (Salamone et al., 2021; Canales-Johnson et al., 2015; García-Cordero et al., 2016; Salamone et al., 2020; Salamone et al., 2018). The interoceptive condition was administered without any feedback to obtain an objective measure of interoceptive accuracy (Salamone et al., 2021; Garfinkel et al., 2015).

After the priming phase (interoceptive or exteroceptive), the participants performed the FER task. In this phase, the participants had to recognize whether the facial emotion was neutral, positive or negative. The stimuli were presented on the basis of Ekman and Friesen’s images (Ekman and Friesen, 1971) in 4 counterbalanced blocks per participant. Fifty-six faces were presented in a pseudorandom order, with 8 neutral, 16 positive (8 happiness, 8 surprise), and 32 negative faces (8 anger, 8 disgust, 8 sadness, 8 fear) (Salamone et al., 2021) (Supplementary Figure 1).

### 2.3 Statistical analysis

#### 2.3.1 Behavioral analysis

FER was performed via the inverse efficiency score (IES) (Salamone et al., 2021), which combines reaction time (RT) and accuracy to analyze weighted behavioral outcomes (Jacquet and Avenanti, 2015). The IES was calculated by dividing the RT index by the percentage of correct responses to control for biases due to reaction times with low accuracy (Salamone et al., 2021; Brozzoli et al., 2008; Mevorach et al., 2006). This means that higher IES scores imply worse performance. The average IES was determined from the two FER blocks following the interoceptive condition and the two blocks following the exteroceptive condition per subject for each type of emotion in the negative, positive, and neutral conditions (Supplementary material 1.4, Statistical analysis).

#### 2.3.2 High-density EEG (HD-EEG) preprocessing and analysis

For interoceptive priming recording, the Biosemi® Active-Two system with 128 channels at 1024 Hz + 2 external adhesive Ag/Ag-Cl electrodes placed on the left lower abdominal quadrant and under the right clavicle for ECG acquisition, as well as two electrodes placed on the left and right mastoids for reference fixation, were used (Ventura-Bort and Weymar, 2024; Migeot et al., 2023). The data sampling rate was changed to 256 Hz and filtered between 0.5–40 mV. Eye movement correction was performed via independent component analysis (Kim and Kim, 2012) and a visual inspection protocol following the parameters of previous studies (Salamone et al., 2021; García-Cordero et al., 2016; Yoris et al., 2018). The ECG signal and R waves were identified with the peakfinder function in MATLAB (Yoder, 2009). This signal was segmented for HEP analysis into epochs from −200 to 800 ms (Canales-Johnson et al., 2015; García-Cordero et al., 2016; Salamone et al., 2020; Yoris et al., 2018). HEPs were generated for each condition in an 800 ms window, delimiting EEG epochs between −200 and 500 ms close to the R-wave peak, corrected between −300 and 0 ms (Salamone et al., 2021; Salamone et al., 2018). HEPs were generated for each condition prior to FER (interoception-exteroception). Thus, to test for HEP modulations in the PTSD group, the interoception conditions were compared through a point-by-point Monte Carlo permutation test with bootstrapping (5,000 permutations, *p* = < 0.05) (Manly, 2018). The main HEP analyses were conducted on a frontal ROI associated with interoceptive attention modulation (Couto et al., 2015; Salamone et al., 2021; García-Cordero et al., 2016), which is composed of 11 electrodes: C9, C10, C14, C15, C18, C19, C20, C27, C28, C31 and C32. Analyses were also performed with four frontal ROIs: left-frontal: C26, C27, C28, C31, C32, D3, D4, D5, D6, and D7; central-frontal: C11, C12, C18, C19, C20, C21, C22, C23, C24, and C25; right-frontal-lateral: B17, B18, B19, B20, B21, B22, B23, B30, B1, and B2; and left-frontal-lateral: D11, D12, D13, D14, D17, D18, D19, D20, D27, and D28, to assess modulation at different locations.

## 3 Results

### 3.1 Demographics and cognitive data

Significant differences were found in the IFS and MoCA test scores between the groups (including working memory scales) (Table 1).

### 3.2 FER results

We found differences in FER across groups in the positive, negative, and neutral conditions (*F* = 5.653, *p* = 0.0068) (Supplementary Figure 1). Contrasts revealed that both the CPTSD group and the PTSD group performed worse than the CG did (CPTSD: mean = 1.037, SD = 0.718; PTSD: mean = 0.904, SD = 0.632; CG: mean = 0.609, SD = 0.516; η^2^ = 0.22). Additionally, comparisons by group and priming type did not reveal differences between the interoception and exteroception conditions (*F* = 0.692, *p* = 0.405) (Supplementary material 2.1, FER results).

### 3.3 Interoceptive and exteroceptive accuracy

In addition, we found differences between groups in interoceptive and exteroceptive accuracy. More specifically, the contrast showed bordering differences in the interoceptive and exteroceptive conditions between the PTSD patients and the CG, and the PTSD patients and the CG also presented significant outcomes in the exteroceptive conditions, as expected (Supplementary Table 2).

### 3.4 Correlations between interoceptive accuracy and emotion recognition

The correlations between interoceptive accuracy and the emotion recognition index indicated that a higher interoceptive accuracy index was associated with better performance in recognizing negative emotion in the CG (*R* = −0.66, *p* = 0.004) (Supplementary Figure 2 and Supplementary Table 3). However, this relationship was not observed in the CPTSD and PTSD groups (*R* = −0.4, *p* = 0.29; *R* = −0.39, *p* = 0.096, respectively). Furthermor, exteroceptive accuracy did not show any significant associations in any of the group (Supplementary Figure 2).

Separate correlations for positive and neutral emotions revealed no significant relationships between interoceptive accuracy and emotion recognition in any of the groups (Supplementary Figure 2 and Supplementary Table 3). When combining the CPTSD and PTSD groups, victims collectively performed better on the IES in interoceptive priming, as compared to the correlation observed between the IES and accuracy in exteroceptive priming (Supplementary Figure 2).

Moreover, interoceptive accuracy emerged as a significant predictor of high performance in the FER of negative emotions in the CG (*β* = −6.12, *p* = 0.004), accounting for 39% of the variance. Similarly, in the group of victims, interoceptive accuracy significantly influenced FER performance in response to negative emotions (*β* = −3.46, *p* = 0.008). This effect may be partially explained by the inclusion of individuals with PTSD in this group, with interoceptive accuracy accounting for 20% of the variance (R^2^ = 0.20).

### 3.5 EEG results

**Figure 1:**
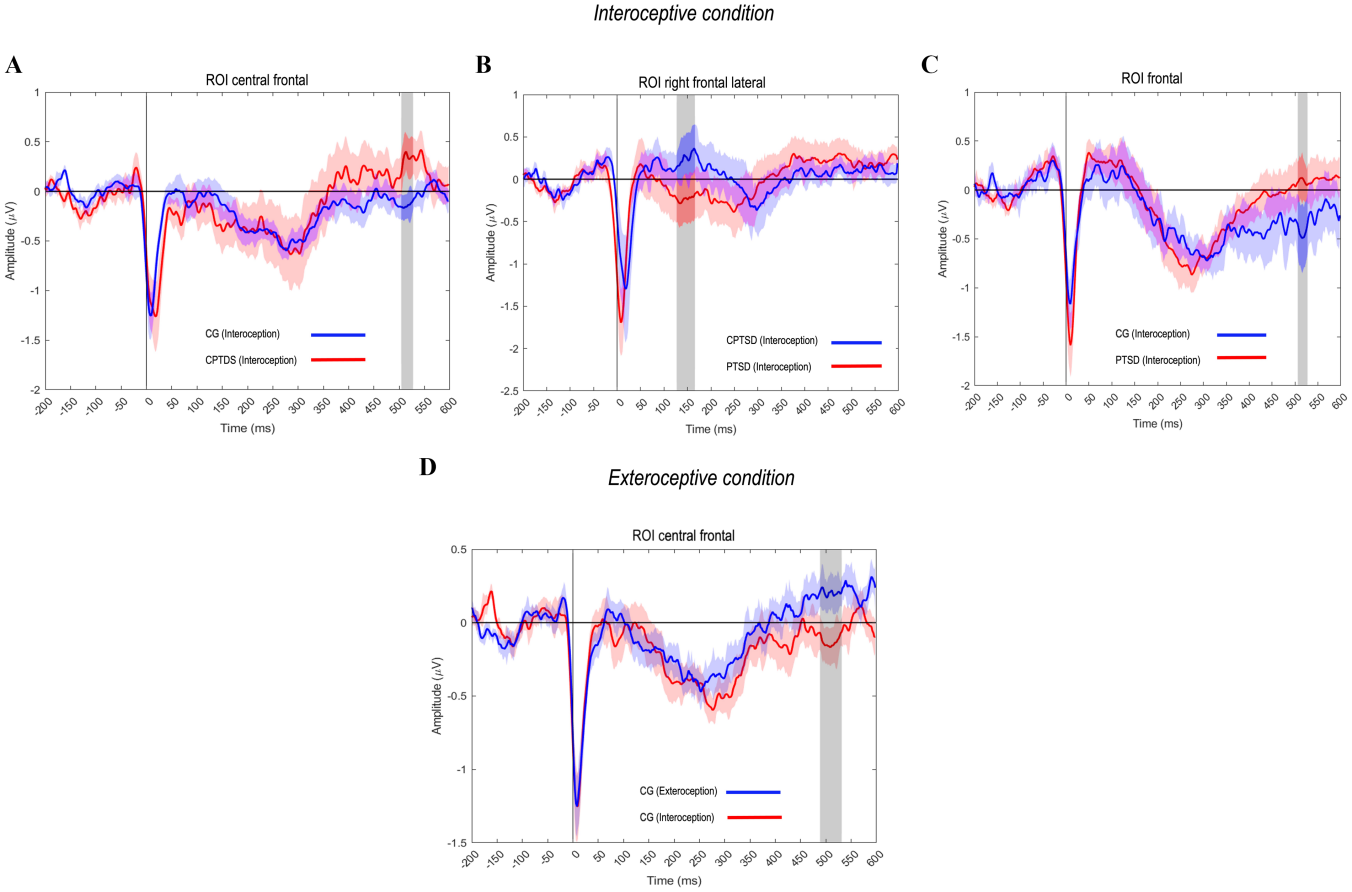
HEP amplitude modulations across groups and conditions. **(A)** The CPTSD group showed significantly greater (less negative) HEP amplitudes in the interoceptive condition at 500 ms in the fronto-central ROI, reflecting a second HEP component (250–600 ms) linked to cognitive processing of the sensory stimulus. **(B)** Significant differences between the CPTSD and PTSD groups were observed in the right frontal-lateral ROI at 150 ms, particularly in the first HEP component (50–250 ms), suggesting heightened early afferent processing from the heart to the brain in the victim groups. **(C)** The PTSD and CG groups exhibited significant differences in the second HEP component at 500 ms, with the PTSD group showing less negative HEP amplitudes. **(D)** Significant differences in the frontal region between the interoceptive and exteroceptive conditions in the CG at 500 ms, indicating a second HEP component, potentially associated with blood pressure waves synchronized with brain activity.

Compared to the CG using an unpaired t test, the CPTSD group showed significantly greater (i.e., less negative) HEP amplitudes in the interoceptive condition, particularly in the fronto-central region of interest (ROI) at 500 ms [M = −0.108, SD = 0.485 for CPTSD-Intero; M = −0.121, SD = 0.472 for CG-Intero; *t*(16.567) = −2.94325, *p* = <0.01, *d* = 0.022] (Figure 1A). This finding revealed differences in a second HEP component (250–600 ms), in which the CPTSD group showed higher amplitude than the CG, suggesting hyperactivity in the cognitive processing of the sensory stimulus. We also found significant differences between the CPTSD and PTSD groups in the right frontal–lateral ROI at 150 ms [M = −0.002, SD = 0.507 for CPTSD-Intero; M = −0.022, SD = 0.527 for PTSD-Intero; *t*(13.439) = −3.027, *p* = 0.0132, *d* = 0.040], specifically, in a first HEP component (50–250 ms). In this window, the CPTSD group again showed less negative (i.e., larger) amplitudes than the PTSD group, suggesting increased early afferent processing from the heart to the brain in CPTSD (Figure 1B).

Additionally, PTSD and CG groups showed significant differences in a second HEP component at 500 ms (Figure 1C), with the PTSD group exhibiting less negative HEP amplitudes than the CG [M = −0.121, SD = 0.696 for PTSD-Intero; M = −0.178, SD = 0.626 for CG-Intero; *t*(30.936) = 2.392, *p* = < 0.02, *d* = 0.086], indicating stronger interoceptive cortical responses in the PTSD group. Finally, as anticipated, significant differences were found in the paired t test in the frontal region between the interoceptive and exteroceptive conditions in the CG at 500 ms [M = −0.121, SD = 0.472 for CG-Intero; M = −0.062, SD = 0.507 for CG-Extero; *t*(16) = −1.910, *p* = 0.015, *d* = 0.119]. This difference likely reflects a second HEP component, potentially related to blood pressure waves synchronized with brain activity, with less negative and wide amplitudes related to the exteroceptive event, in contrast to what we found with the group of victims (CPTSD-PTSD) (Figure 1D).

## 4 Discussion

To our knowledge, this is the first study to investigate FER using interoceptive priming tasks and cardiac interoceptive signals measured by HEP amplitudes in victims of armed conflict in Colombia with PTSD symptoms and healthy controls. Behaviorally, the observed FER impairments indicate that the PTSD and CPTSD groups exhibited deficits in FER. Specifically, compared with those in the CG and PTSD, the performance of CPTSD victims in FER decreased after receiving interoceptive priming versus exteroceptive priming. Compared with the controls, the subjects with CPTSD exhibited a reduced amplitude of the HEP in the frontocentral regions during interoceptive processing. Significant differences were observed between the CPTSD and PTSD groups in the right frontal–lateral region during interoceptive priming. Finally, the associations between interoceptive-exteroceptive accuracy and the IES revealed that the CG improved the FER accuracy of negative emotions in interoceptive priming, and we observed this same result in the correlations with grouped victims. Despite this, victims did not increase their accuracy in exteroceptive priming.

### 4.1 Interoceptive priming on emotion recognition

The performance of healthy controls with respect to negative emotions was lower during interoceptive priming. This finding could be linked to the influence of embodied congruency, described in studies that included heart rate and demonstrated a facilitating effect on the emotional processing of negative emotions such as fearful faces, but no such effect was observed for disgusted or neutral faces (Pezzulo et al., 2018). This approach suggests that a physiological condition such as a heartbeat is an interoceptive element that allows the simulation of certain emotions, but favors the exteroceptive processing of emotional stimuli (Yu et al., 2021) and possibly interferes with FER. Despite this support, it is unclear how interoceptive priming could induce feelings that are not congruent with negative stimuli presented in the task. In addition, we found deficits in FER general conditions in PTSD and CPTSD victims compared with controls, as expected in previous PTSD studies (Castro-Vale et al., 2020; Williams et al., 2018). After interoceptive priming, the FER of negative emotions did not improve in participants with CPTSD, whereas victims with PTSD **showed** better performance according to the IES index. These findings suggest that interoceptive priming differentially affects the FER of negative emotions in the presence of PTSD or CPTSD. These results indicate that alterations in interoceptive predictive coding mechanisms are linked to abnormal emotional processing and basic emotions in PTSD (Passardi et al., 2019; Couette et al., 2022; Wang et al., 2016; Couette et al., 2020). Additionally, these observed differences could be due to criteria related to the diagnosis of CPTSD, which has a set of symptoms called disturbances in self-organization (DSO) (Fernández-Fillol et al., 2021; Ford, 2021). These symptoms include affective dysregulation; therefore, it is plausible that interoceptive priming improved FER performance in the PTSD group, but did not favor the CPTSD group because of the nature of the symptoms. Moreover, trauma-related symptoms impact the recognition of basic emotions, as exposure to violence affects emotional processing in adulthood (Umiltà et al., 2013). The observed differences between the PTSD and CPTSD groups may suggest that changes in self-organization are linked to significant and sustained exposure to traumatic events and interpersonal challenges during early development (Hyland et al., 2017). An example of this is intergenerational exposure to the conflict in Colombia, which has lasted for over six decades. We interpret these findings within the SMH (Damsio, 1994; Damasio, 1999) frameworks and the SAME (Cacioppo et al., 1992), suggesting that emotions depend on how the brain interprets interoceptive signals. Therefore, disruptions in these interoceptive signals may significantly impair emotional regulation, as occurs in disorders such as PTSD. Theoretically, this is consistent with the idea that incongruence between internal and external states may hinder the value assigned to cognitive or emotional actions (Ochsner and Gross, 2014). Disruptions between physiological and affective components and exaggerated threat detection may impair emotion identification (Brown et al., 2020). Recent proposals (Silvanto and Nagai, 2025) have suggested a relationship between interoception and mental imagery, emphasizing the critical role of the insular cortex and anterior cingulate cortex (ACC) in integrating bodily and emotional processes. Specifically, the ACC serves as an integrative hub that combines sensory, emotional, and cognitive inputs during perception. This integration is particularly relevant in PTSD, where flashbacks represent a core symptom. Given this association, future research should investigate how interoceptive activation influences mental imagery and subsequently impacts FER in populations with PTSD and CPTSD. The connection with internal sensations increases CPTSD symptoms, which can be explained by generative models that combine exteroceptive “threat” and interoceptive “allostasis” priors (Krupnik, 2020; Wilkinson et al., 2017). In this sense, functions may be more affected in victims with CPTSD than in victims with PTSD because allostatic-interoceptive prediction requires regulating the body’s internal environment to predict and meet the needs generated by environmental demands before they arise (Migeot et al., 2022). This hypothesis should be explored in future studies.

According to evidence from a neurobiological model, these deficits could be explained by hyperactivation of the amygdala and insula in CPTSD (Suarez-Jimenez et al., 2020; Ressler et al., 2022) and deficits in the recognition of negative emotions (Passardi et al., 2019; Greene et al., 2020). Conversely, interoceptive accuracy was negatively correlated with the IES for negative emotion recognition in subjects not exposed to conflict and with the CPTSD score. Enhanced interoceptive ability improves emotion recognition, highlighting that interoceptive accuracy is closely related to the peripheral processing of emotional stimuli (Calì et al., 2015; Herbert et al., 2007; Pollatos et al., 2007). This evidence suggests that central and peripheral pathways are affected in CPTSD and are affected by exposure to violence. This hypothesis supports the idea that interoceptive accuracy increases sensitivity to emotional recognition of negative stimuli in healthy controls (Salamone et al., 2021; Critchley and Garfinkel, 2017; Terasawa et al., 2014) and in populations with emotion recognition deficits, such as the PTSD group.

### 4.2 Electrophysiological markers: HEP

With respect to electrophysiological markers, we found that, in contrast with controls, PTSD and CPTSD victims presented attenuated negative amplitudes in HEPs in frontocentral regions during interoceptive processing. This result contrasts with a previous study that did not show differences in HEP amplitudes between PTSD patients and controls (Schmitz et al., 2021). This finding suggests that this condition is more typical of neuropsychiatric disorders, as demonstrated in other studies (Flasbeck et al., 2020; Pang et al., 2019; García-Cordero et al., 2016) and in older adults (Kamp et al., 2021). Additionally, attenuated HEP amplitudes are typical of disorders with high prevalence rates of early-life maltreatment (Schmitz et al., 2020; Mueller et al., 2015). This may be the case for the older adult community we examined, who have been subjected to repeated conflicts over an extended period of time, resulting in CPTSD symptoms. Future studies should examine the effects of early-life maltreatment on interoception in victims of violence, as prior research suggests that such maltreatment may increase the risk of stress-induced interoceptive dysfunction (Schaan et al., 2019; Schulz et al., 2022).

### 4.3 Limitations and future directions

The present study has several limitations. First, the sample size was small, particularly in the CPTSD group, with the majority of participants being women meticulously matched with healthy controls. Although previous studies have acknowledged these factors, the low proportion of men and the small sample size (Gómez et al., 2024; Gómez et al., 2022) may partly explain these findings. The study employed a rigorous selection process with strict inclusion and exclusion criteria, ensuring the absence of neurological and psychiatric disorders, which are common in individuals affected by Colombian violence. Most participants were female survivors of violence, with men having the highest mortality rates (Urdinola et al., 2017). Research has shown that victims experience profound and lasting psychiatric and neurological difficulties as a result of the war. Another potential limitation relates to the age distribution of the subjects. Although the variations observed in interoception, FER, and HEP are not explained by the low performance in cognitive assessments (e.g., MOCA and IFS) in PTSD and CPTSD, caution should be exercised when extrapolating the results of the present study. Many variables related to biological and social factors may influence the context of violence to which the Colombian population has been exposed in recent decades. Future studies should include a larger number of subjects and consider the possibility of including other mental disorders, as these could also result from violence. Additionally, incorporating assessments of mental imagery would further clarify its role in emotional recognition and interoceptive mechanisms among populations with PTSD and CPTSD complex PTSD, given that interoceptive processing and mental imagery share common neural substrates, particularly the insular cortex. Investigating the relationships among aging, potential cognitive disorders, and social determinants associated with violence is crucial. In addition, research should include younger subjects to study the effects of violence on interoception and social cognition.

## 5 Conclusion

The study demonstrated that interoception increases the ability to discern negative emotions in individuals with PTSD but not in those with CPTSD. Additionally, interoception enhanced the perception of neutral emotions across all groups. Electroencephalographic (EEG) analysis revealed theta-band oscillations (HEPs), particularly in frontal and frontocentral regions. These results highlight the importance of interoceptive training in enhancing emotion identification, especially in individuals with PTSD. The potential implications of these findings for treating PTSD and CPTSD are substantial. Future research should explore the role of DSO in FER and interoception among individuals affected by armed conflict with PTSD and PTSD-CPTSD. Ultimately, this improved understanding will facilitate the development of more effective treatments. The findings of this study are particularly relevant, as it is among the first conducted within the Colombian population, offering valuable insights into how prolonged exposure to armed conflict affects interoceptive processing and emotional recognition in individuals diagnosed with PTSD.

## Data Availability

The original contributions presented in the study are included in the article/Supplementary material, further inquiries can be directed to the corresponding authors.
