# Supplementary material for: Impaired interoception in Colombian victims of armed conflict with PTSD: a preliminary HEP study
Source: Front Psychol. 2025 Apr 25;16:1567574. doi: 10.3389/fpsyg.2025.1567574 (PMC12061942; doi:10.3389/fpsyg.2025.1567574)
Supplement: Supplementary file 1 [file Table_1.docx]

Supplementary Material

*Impaired interoception in Colombian victims of armed conflict with PTSD: A preliminary HEP study*

**Eduar Herrera** **^1^****^*^, Daniela Gutierrez Sterling ^2^†, Alvaro Barrera-Ocampo^3^** **Juliana Orozco Jaramillo ^4,^ Hernando Santamaría-García, ^5,6^ Agustina Birba ^7,8*^**

*** Correspondence:** Eduar Herrera
[eherrera@icesi.edu.co](mailto:eherrera@icesi.edu.co)

Agustina Birba

[agustina.birba@gmail.com](mailto:agustina.birba@gmail.com)

# Supplementary Materials and Methods

- 1. ***International Trauma Questionnaire (ITQ) Scores***

| ***Suppl. Table 1 : International Trauma Questionnaire (ITQ) Scores*** | | | | | | |
| --- | --- | --- | --- | --- | --- | --- |
| ***Group*** | ***ITQ score*** | ***SE*** | ***IC (95%)*** | ***F*** | ***p*** | ***Contrasts*** |
| CG | 0 (-) | 0 | - | 107.62 | < 0.0005*** | *CG vs. PTSD*: *p* = < 0.0001*** |
| PTSD | 8.52 (5.61) | 2.704 | 5.821 – 11.230 |  | *η*p^2^ = 0.84 | *CPTSD vs. PTSD*: *p* = < 0.0001*** |
| CPTSD | 27.75 (5.75) | 4.807 | 22.942 – 32.557 |  |  | *CG vs. CPTSD*: *p* = < 0.0001*** |
| The values reported correspond to the mean and standard deviation (SD). SE: Standard error. IC: Confidence intervals (95%). Effect size partial eta-squared (*η*p^2^).  ***Variable with significant differences (p <0.005). | | | | | | |

***1.2 Clinical measures***

*Sociodemographic and clinical questionnaire*

This brief semistructured interview collected information related to demographic data, family history of illness, history of medication and psychoactive substance use, and the presence of neurological, psychiatric, and chronic illnesses.

*The Montreal Cognitive Assessment (MoCA)*

It is a cognitive screening instrument for mild cognitive impairment and dementia with tasks that assess attention, executive functions, language, memory, visual-constructive abilities, calculation, and orientation (1). It has a maximum score of 30 points and takes 10 minutes to administer. The Colombian validation of this instrument has a reliability coefficient of .086 in a population with a low educational level (2)

*Ineco Frontal Screening (IFS)*

It is a screening test designed to assess frontal dysfunction. The tasks assess motor programming, contradictory instructions, abstraction ability, spatial working memory and inhibitory control. It has a reliability coefficient of .080 (3)

*Hopkins Symptom Checklist-58 (HSCL-58)*

It is a 25-item scale with 2 dimensions to assess anxiety and depression with a Likert-type scale from 1 to 4, where 1 corresponds to "not at all" and 4 corresponds to "very much"(4). The score is calculated as the average score of all items.

***1.3 Task and performance on a beat detection***


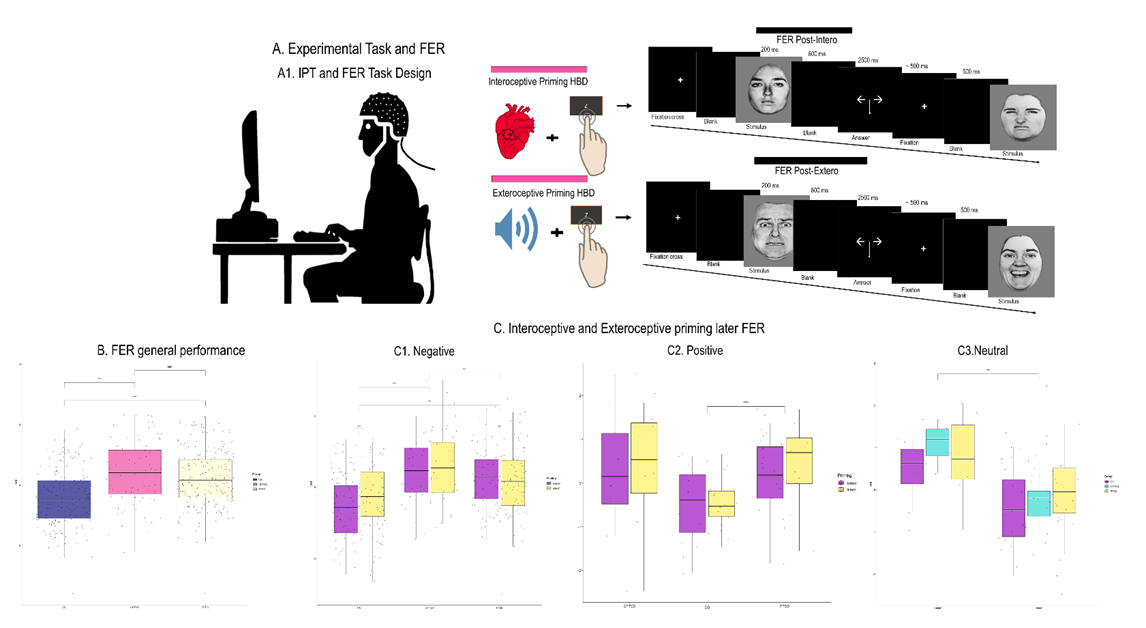


**Supplementary Figure 1.** **(A)**. IPT task design (Salamone, et.al, 2021). The first graph represents the interoceptive priming paradigm. **(A1)** Participants performed the task accompanied by a researcher in front of the computer simultaneously with the hd-EEG recording. The Priming phase was composed of two phases: an interoceptive and an exteroceptive phase. Each condition was presented twice. The phases were counterbalanced by each participant (pink bars), interspersed by the emotional recognition phase (black bars). The response options for the emotion recognition phase were determined as Negative, Positive, and Neutral by pressing the corresponding scroll keys (left, right, and center, respectively). **(B** and **C)** We assessed participants' performance on a beat detection task and compared IES (y-axis) to groups (x-axis) using a repeated-measures ANOVA. Better performance is characterized by higher accuracy scores. Significant results are identified with an asterisk (*), p < 0.05.

*(This supplementary figure is available in a PowerPoint editable slide called “Suppl. Figure_1”)*

***1.4 Statistical analysiss***

To determine the interoceptive accuracy index, the mean difference between the frequency of responses and the underlying heart rate in different time windows was calculated via the equation (5).

ANOVA factorial designs were used to identify differences in FER and interoceptive priming between groups. To assess emotion recognition deficits across groups, we performed a one-way ANOVA with a three-level between-subject factor (Group: CG, PTSD and CPTSD) as the independent variable and the IES score as the dependent variable. Two-factor ANOVA was subsequently applied for each type of emotion separately, again with the within-subject factor of priming type and the between-subject factor of Group. Post hoc analyses were performed specifically pairwise (CG-CPTSD, CG-PTSD, and PTSD-CPTSD) with Tukey's HSD tests. Nonnormalized data analyses may generate Type I and II errors(6). To avoid this situation, IES scores were normalized via Box–Cox and order–norm transformation, guaranteeing the assumptions of normality, linearity, and homoscedasticity (7), and the reported means and confidence intervals were back-transformed to the original scale for interpretation (8). Likewise, outliers were treated by eliminating outliers at 1.5 of the IQ range (9). Additionally, to avoid biased interpretations of the IES and FER results, dependent variables such as depression and anxiety were controlled and covaried per subject in each group. Qualitative data were processed from nominal measurement scales as measures of central tendency (mean and standard deviation) and chi-square (χ2) tests. The data were systematized in Microsoft Excel and analyzed via RStudio v. 1.1.383 (10). To explore the associations between interoceptive accuracy and the IES score for each group, Pearson's r parametric correlation tests were performed with normalized data and with outlier treatment. The results with a statistical cutoff of p < 0.05 were considered significant.

1. ***Results***

***2.1 FER results***

ANOVA of the effects of the FER task on negative emotions revealed a significant interaction effect between group and priming type (*F*= 6.15, *MS*= 1.60, *p* = 0.022) **.**Post hoc analysis revealed differences between interoceptive priming and exteroceptive priming in the CG in terms of the FER of negative emotions (CG postintervention: mean= 0.570, SD= 0.382; CG postextraction: mean= 0.457, SD= 0.346; Tukey HSD, *df*= 319, *p =* 0.028). The PTSD group performed better in recognizing negative emotion after receiving interoceptive priming than after receiving exteroceptive priming (PTSD Post-Intero: Mean= 0.765, SD = 0.487; PTSD Post-Extero: Mean = 0.856, SD = 0.600; *df=* 47.6, *p =* 0.04). Moreover, after the exteroceptive priming phase, the PTSD group performed poorly in contrast to the CG (*df=* 47.6, *p* = 0.004), but following the interoceptive priming phase, their performance was not significantly different from that of the control group (PTSD Post-Intero: Mean= 0.765, SD = 0.487; CG Post-Intero: Mean = 0.570, SD= 0.382; *df*= 51.0, *p*= 0.129). In other words, interoceptive priming improved the FER of negative emotions in the PTSD group, eliminating the significant differences observed after exteroceptive priming with respect to the control group (Suppl. Figure 1C1).

In contrast, compared with the CG, the CPTSD group had impairments in the FER task after both interoceptive priming (CPTSD Post-Intero: Mean= 1.077, SD= 0.711; CG Post-Intero: Mean= 0.570, SD= 0.382; Tukey HSD*, df=* 49.9, *p* = 0.041) and exteroceptive priming (CPTSD Post-Extero: Mean= 0.948, SD= 0.569; CG Post-Extero: Mean= 0.457, SD= 0.346; Tukey HSD*, df*= 46.5, *p* = 0.0124, *d*= -1.403). Interoceptive priming negatively affected the FER of negative emotions, as evidenced by lower performance (CPTSD Post-Intero: Mean= 1.077, SD= 0.711; *df*= 49.9, *p* = 0.041), than did exteroceptive priming (CPTSD Post-Extero: Mean = 0.948, SD= 0.569; *df*= 46.5, *p* = 0.012). Finally, the PTSD group did not show significant differences in the type of priming received with respect to the CG after both the interoceptive phase (*df*= 48.6, *p*= 0.163, *d*= -1.001) and the exteroceptive phase (*df*= 46.5, *p*= 0.123).

Differences between groups were observed in the FER of positive emotions (*F*= 4.57, *p=* 0.016) (Supplementary data, Suppl. Figure 1C2). Post hoc contrasts revealed differences in FER performance between the PTSD group and the CG in the phases following interoceptive priming compared with exteroceptive priming (PTSD postinterop: mean= 0.735, SD= 0.440; CG postinterop: mean= 0.335, SD = 0.167; Tukey HSD, *df=* 62.1, *p=* 0.025, *d*= -1.053). Specifically, the CG’s performance in FER to positive emotions was compromised after exteroceptive priming but improved after interoceptive priming, indicating that interoceptive priming favorably impacts positive emotion processing in controls.

In the CPTSD group, compared with exteroceptive priming, interoceptive priming unfavorably affected FER performance for positive emotions (CPTSD Post-Intero: Mean= 0.900, SD= 0.806; CPTSD Post-Extero: Mean= 1.024, SD = 1.141; Tukey HSD, *df=* 33.4, *p=* 0.838). The same pattern was observed in the PTSD group (PTSD postintervention: mean= 0.735, SD= 0.440; PTSD postintervention: mean = 0.555, SD= 0.316; *df*= 37.1, *p*= 0.196) (Suppl. Figure 1C2). This suggests that no significant differences were observed in the recognition of positive emotions according to the type of priming received (interoceptive or exteroceptive). Finally, regarding neutral emotions, a main effect of priming type was found (*F =*11.3, *MS=*9.43, *p=* 0.001), indicating that all groups improved after interoceptive priming (Suppl. Figure 1C3).

***2.1.1 Depression, anxiety and cognitive function effects on FER***

When controlling for depression levels, we still observed differences in performance between groups in FER performance in the positive, negative, and neutral conditions (*F*= 4.428, *p* = 0.018, η^2^ =0.05), and we found that the CPTSD and PTSD groups presented lower FER performance than the controls did, with no interaction effect between depression and anxiety. Additionally, analysis of the covariate MoCA revealed no influence on the FER model (*F=*0.0996*, p=* 0.752, η^2^ = <0.01).

Controlling for depression scores in the negative condition yielded similar results, with a significant interaction between group and priming type (*F*= 3.77, *MS*= 1.59, *p*= 0.023, η^2^ = < 0.01).

The CG showed better performance under both the interoceptive and exteroceptive conditions (CG PostIntero: mean = 0.570, SD= 0.382; CG Postextero: mean= 0.457, SD= 0.346; Tukey HSD*, df*= 318, *p* = 0.028). The analyses of FER for negative emotions controlling for depression scores also revealed that the PTSD group’s performance was lower than that of the CG (*df=* 46.5, *p*= 0.007). Additionally, FER analyses for negative emotions, controlling for MoCA scores, revealed differences in the CG under interoceptive and exteroceptive priming (*df*= 320, *p*= 0.019).

In addition, the analysis of covariance with the scores of depression and anxiety in positive emotions revealed differences between groups (*F=*4.77, *MS=* 2.53*, p=* 0.014), specifically, differences in performance between the CG and PTSD in the interoceptive priming versus the exteroceptive priming (Tukey HSD*, df=* 61, *p=*0.02). However, a significant interaction effect was found between groups and MoCA performance for positive emotions (*F*= 5.359, *MS*= 2.768, *p*= 0.009, η^2^ = 0.23). Post hoc analyses revealed differences between the CG and PTSD (Tukey HSD, *df=* 34.8, *p=* 0.0281, *d*= -1.29), and adding the type of priming provided that we observed a main effect of the MoCA on FER in positive emotions (*F=* 7.757, *MS*= 3.257, *p*= 0.007, η^2^ = 0.15), specifically in the comparison between Intero and Extero priming in the CG (Tukey HSD, *df=* 320, *p=* 0.019).

Finally, depression and anxiety scores for neutral emotions did not influence the main effect of the type of priming received (*F=* 14.177, *MS=* 5.393, *p=* 0.0003, η^2^ = 0.03) or the improvement of the groups after interoceptive priming (Tukey HSD, CG: *df=* 58.1, *p=* 0.119; PTSD: *df=* 42.8, *p=* 0.045; CPTSD: *df=* 48.4, *p=* 0.036). In addition, the covariance of the MoCA with FER performance in neutral emotions showed a main effect of this variable (*F=* 14.002, *MS=* 1.839, *p=* 0.0005, η^2^ = 0.26) without influencing FER performance compared by group and by type of priming (Tukey HSD, CG Intero-Extero: *df=* 320, *p=* 0.119; PTSD Intero-Extero: *df=* 317, *p=* 0.187; CPTSD Intero-Extero: *df=* 317, *p=* 0.270).

***2.1.2 Interoceptive and exteroceptive accuracy***

| ***Suppl. Table 2: Behavioral results of interoceptive and exteroceptive accuracy.*** | | | | |  |
| --- | --- | --- | --- | --- | --- |
| ***Variable*** | ***CG*** | ***PTSD*** | ***CPTSD*** | ***Statistical results*** | ***Contrasts*** |
| Interoceptive accuracy | 0.179 (0.940) | -0.094 (0.912) | -0.214 (0.891) | *F* = 3.829 | *CG vs. PTSD: p =* 0.082 |
|  |  |  |  | *p* = 0.028* | *CG vs. CPTSD: p =* 0.041* |
|  |  |  |  | *η*p^2^= 0.14 | *PTSD vs. CPTSD: p =* 0.716 |
|  |  |  |  |  |  |
| Exteroceptive accuracy | 0.187 (0.921) | -0.073 (0.931) | -0.293 (0.888) | *F =* 5.061 | *CG vs. PTSD: p =* 0.116 |
|  |  |  |  | *p =* 0.010* | *CG vs. CPTSD: p =* 0.035* |
|  |  |  |  | *η*p^2^= 0.18 | *PTSD vs. CPTSD: p =* 0.565 |
| The values reported correspond to the mean and standard deviation (SD). Effect size partial eta-squared (*η*p^2^).  *Variable with significant differences (p= < 0.05). | | | | | |

***2.1.3 1 Correlations between interoceptive accuracy and emotion recognition***

**
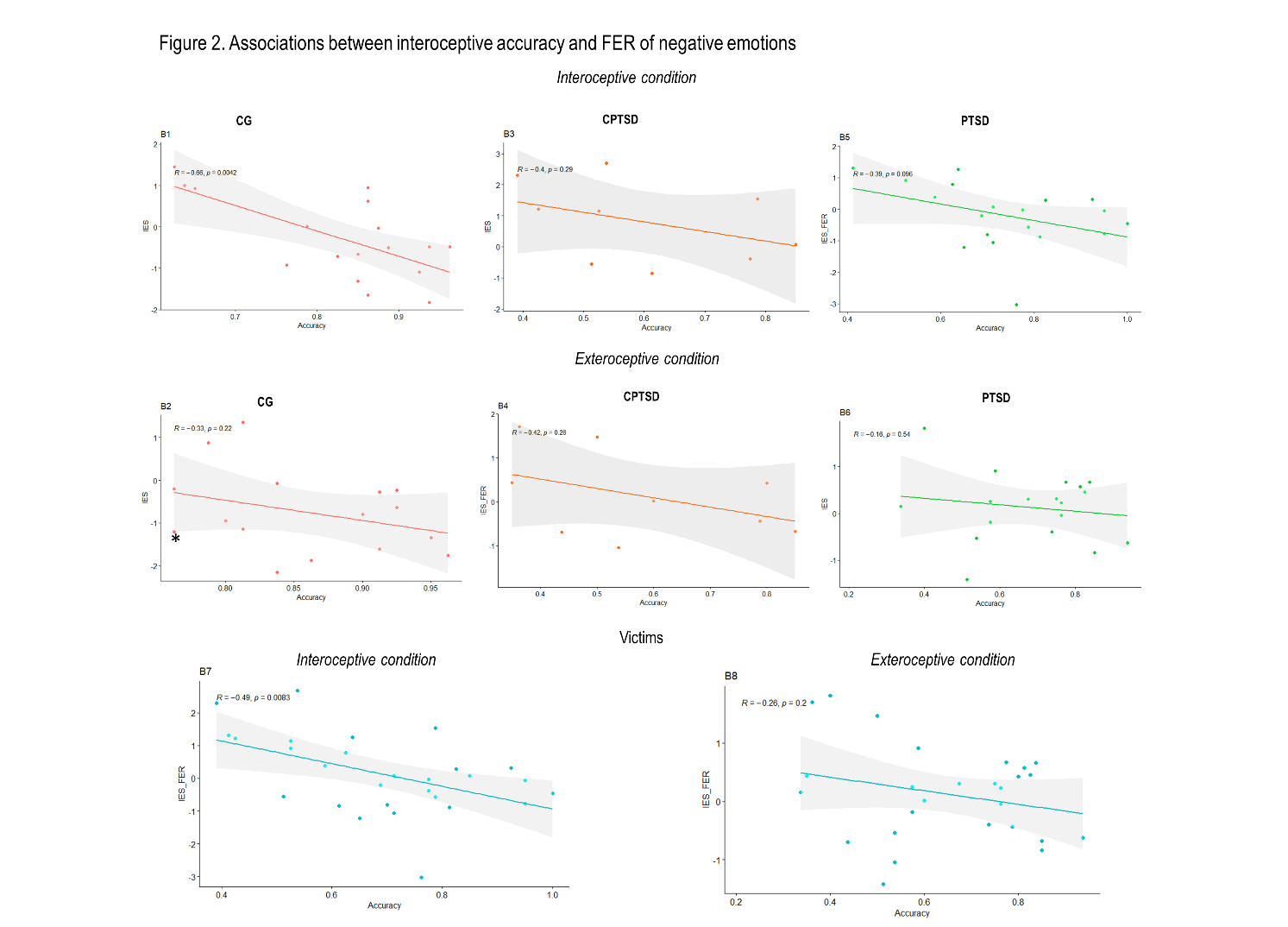
**

**Supplementary Figure 2. Associations between interoceptive accuracy and FER of negative emotions.** Correlation results: To identify associations between the time estimate of interoceptive accuracy (accuracy) and the negative emotion FER index (IES_FER), a Pearson's R correlation coefficient was applied to control participants and victims of armed conflict with PTSD and CPTSD.

*(This supplementary figure is available in a PowerPoint editable slide called “Suppl. Figure_2”)*

| ***Suppl. Table 3: Control correlations between positive and neutral emotion recognition index and interoceptive and exteroceptive accuracy.*** | | | | |
| --- | --- | --- | --- | --- |
| ***Priming type*** | ***Emotion*** | ***Group*** | ***r*** | ***p*** |
| Interoception | Positive | CG | 0.04 | 0.92 |
|  |  | CPTSD | -0.38 | 0.53 |
|  |  | PTSD | 0.15 | 0.60 |
|  |  |  |  |  |
|  | Neutral | CG | -0.06 | 0.86 |
|  |  | CPTSD | -0.17 | 0.83 |
|  |  | PTSD | -0.52 | 0.18 |
|  |  |  |  |  |
| Exteroception | Positive | CG | 0.34 | 0.26 |
|  |  | CPTSD | 0.54 | 0.35 |
|  |  | PTSD | -0.38 | 0.22 |
|  |  |  |  |  |
|  | Neutral | CG | 0.22 | 0.86 |
|  |  | CPTSD | -0.69 | 0.51 |
|  |  | PTSD | -0.06 | 0.89 |
| The data reported corresponds to the Pearson correlation coefficient (r) and p values (p) in interoceptive and exteroceptive condition by groups. | | | | |

**References**

1. Nasreddine ZS, Phillips NA, Bédirian V, Charbonneau S, Whitehead V, Collin I, et al. The Montreal Cognitive Assessment, MoCA: a brief screening tool for mild cognitive impairment. Journal of the American Geriatrics Society. 2005;53(4):695-9.

2. Gómez F, Zunzunegui M, Lord C, Alvarado B, García A. Applicability of the MoCA‐S test in populations with little education in Colombia. International journal of geriatric psychiatry. 2013;28(8):813-20.

3. Torralva T, Roca M, Gleichgerrcht E, Lopez P, Manes F. INECO Frontal Screening (IFS): A brief, sensitive, and specific tool to assess executive functions in dementia–ERRATUM. Journal of the International Neuropsychological Society. 2010;16(5):737-47.

4. Derogatis LR, Lipman RS, Rickels K, Uhlenhuth EH, Covi L. The Hopkins Symptom Checklist (HSCL): A self‐report symptom inventory. Behavioral science. 1974;19(1):1-15.

5. Fittipaldi S, Abrevaya S, de la Fuente A, Pascariello GO, Hesse E, Birba A, et al. A multidimensional and multi-feature framework for cardiac interoception. Neuroimage. 2020;212:116677.

6. Nichols AL, Edlund J. The Cambridge Handbook of Research Methods and Statistics for the Social and Behavioral Sciences: Volume 1: Building a Program of Research: Cambridge University Press; 2023.

7. Osborne J. Improving your data transformations: Applying the Box-Cox transformation. Practical Assessment, Research, and Evaluation. 2010;15(1):12.

8. Salamone PC, Legaz A, Sedeño L, Moguilner S, Fraile-Vazquez M, Campo CG, et al. Interoception primes emotional processing: Multimodal evidence from neurodegeneration. Journal of Neuroscience. 2021;41(19):4276-92.

9. Dash CSK, Behera AK, Dehuri S, Ghosh A. An outliers detection and elimination framework in classification task of data mining. Decision Analytics Journal. 2023;6:100164.

10. Team R. RStudio: integrated development for R. Boston, MA: RStudio. Inc; 2015.
